# Creatine as a Candidate to Prevent Statin Myopathy

**DOI:** 10.3390/biom9090496

**Published:** 2019-09-17

**Authors:** Maurizio Balestrino, Enrico Adriano

**Affiliations:** Department of Neuroscience, Rehabilitation, Ophthalmology, Genetics and Maternal and Child Sciences (DINOGMI), University of Genoa, Largo Daneo 3, 16132 Genova, Italy

**Keywords:** creatine, statin, myopathy, muscle, myalgia, prevention, treatment, pathogenesis, pathophysiology, mitochondria

## Abstract

Statins prevent cardiovascular diseases, yet their use is limited by the muscle disturbances they cause. Rarely, statin-induced myopathy is autoimmune, but more commonly it is due to direct muscle toxicity. Available evidence suggests that statin-induced creatine deficiency might be a major cause of this toxicity, and that creatine supplementation prevents it. Statins inhibit guanidinoacetate methyl transferase (GAMT), the last enzyme in the synthesis of creatine; thus, they decrease its intracellular content. Such decreased content could cause mitochondrial impairment, since creatine is the final acceptor of the phosphate group of adenosine triphosphate (ATP) at the end of mitochondrial oxidative phosphorylation. Decreased cellular synthesis of ATP would follow. Accordingly, ATP synthesis is decreased in statin-treated cells. In vitro, creatine supplementation prevents the opening of the mitochondrial permeability transition pore that is caused by statins. Clinically, creatine administration prevents statin myopathy in statin-intolerant patients. Additional research is warranted to hopefully confirm these findings. However, creatine is widely used by athletes with no adverse events, and has demonstrated to be safe even in double-blind, placebo-controlled trials of elderly individuals. Thus, it should be trialed, under medical supervision, in patients who cannot assume statin due to the occurrence of muscular symptoms.

## 1. Introduction

Inhibitors of the 5-hydroxy-3-methylglutaryl-coenzyme A (HMG-CoA) reductase (“statins”) lower blood cholesterol levels by inhibiting its production in the liver. The rationale for their utilization in human therapy is that, when present in high concentrations, cholesterol enters the arterial wall and becomes an essential factor in the genesis of arteriosclerosis, a major factor in the genesis of cardiovascular diseases [1]. Statins block the hepatic enzyme responsible for cholesterol production, and are therefore essential in reducing the risk of cardiovascular diseases in patients at risk [2]. In addition, they exert additional effects (so called “pleiotropic” effects) that are relatively independent of cholesterol reduction, such as reducing vascular inflammation, decreasing markers of platelet adhesion, reducing oxidative stress, improving endothelial cell function, stabilizing the atherosclerotic plaque, etc. [2,3,4]. By all these various effects, they reduce the progression of arteriosclerosis and the risk of severe cardiovascular accidents, including myocardial infarction and ischemic stroke [5,6,7]. Beside statins, ezetimibe and evolocumab are also available to reduce cholesterol levels; nevertheless, statins remain first choice drugs even in the face of such alternatives [8].

Despite robust evidence of their effectiveness, statins are prescribed less often than they should be [9]. For example, a report showed that statins are not prescribed to 30% of patients that have suffered an ischemic stroke, despite evidence showing their effectiveness in that context [10]. Another report showed that only a minority of patients who were hospitalized after a coronary heart disease event fulfill the guideline recommendation of a high-intensity statin prescription [11]. 

One of the main reasons for the under-prescription of statins is certainly fear of their muscular side effects, the so-called statin myopathy [9,12]. 

Statin-associated muscular symptoms are in fact a well-known side effect of statins. They range from asymptomatic elevation of serum creatine kinase (CK) to life-threatening rhabdomyolysis [13]. In clinical trials, about 1.5–3% of statin users developed myalgia, a percentage that rose to 10–13% in prospective observational studies [14]. In their review, Stroes et al. [13] found muscular symptoms in 7–29% of statin-treated patients, and in a single observational study Bruckert et al. report an incidence of 38% [15]. Statin intolerance is a major cause of patients stopping their consumption and incurring a cardiac event [16].

## 2. Common Hypothesis on Pathogenesis

There is so far no universal consensus on why statin-associated myopathy occurs. Christopher-Stine and Basharat [17] emphasize an immune-mediated mechanism. That, however, is specific to a necrotizing variety of statin-induced myositis, different from the more usual myositis. This very specific autoimmune condition is characterized by the presence of autoantibodies against 3-Hydroxy-3-Methylglutaryl-CoA Reductase (HMGCR), the protein whose gene is inhibited by statins. It is very severe, characterized by muscle necrosis at histology, can occur even years after exposure to statins and is diagnosed by noting the presence of the autoantibodies anti-HMGCR [18]. Mammen considers it “an exceptionally rare side effect of statin use,” and estimates its incidence at “approximately 2 or 3 of every 100,000 patients treated with statins” [19]. This peculiar condition has been reviewed by recent papers, to which we refer the interested reader [18,19,20] while we continue discussing the more frequent, not autoimmune, statin-associated myopathy. 

Despite the fact that many authors have reviewed this subject, the exact mechanisms as to why statins cause muscle toxicity are not known [21,22,23]. Specifically, several intermediates have been proposed as causes of statin-associated myopathy, including the mevalonate pathway and its end products, including non-sterol isoprenoids (farnesol, geranylgeraniol), heme, ubiquinone A, dolichol, squalene, etc. In fact, multiple pathophysiological mechanisms may perhaps contribute to this condition [24]. An extensive review of the pathophysiological mechanisms that have been proposed to explain statin-associated myopathy would be beyond the scope of this paper, so we refer the interested reader to the many fine reviews that have been published so far on this still elusive topic. Nevertheless, Table 1 summarizes some of the most common hypotheses on the pathogenesis of statin-induced (not autoimmune) myopathy that were discussed in the past ten years.

To sum it up, from the above table we can conclude that not only the exact molecular pathogenesis of statin-induced myopathy is still unknown, but also several mechanisms have been hypothesized, including altered statin pharmacokinetics, mitochondrial toxicity, apoptosis, impaired muscle regeneration, etc. Some of the proposed mechanisms that may cause statin-induced myopathy are related to energy metabolism and, in particular, to creatine metabolism.

## 3. Statins Decrease Creatine Synthesis

Creatine is of paramount importance to normal muscle function [31,32,33]. It is obtained through the diet, but it is also synthesized by the body [34]. Under normal conditions, both pathways are active in maintaining appropriate concentrations of tissue creatine, but when creatine synthesis is impaired, only the dietary source remains. Under such conditions of blocked creatine synthesis, the usual intake of creatine with the diet may not be sufficient to meet the body’s requirements. This is very clear from the rare hereditary diseases where creatine synthesis is impossible due to the malfunctioning of either l-Arginine:glycine amidinotransferase (less commonly known as “glycine amidinotransferase, mitochondrial”) (AGAT or, less commonly, GATM) or Guanidinoacetate methyltransferase (GAMT), the two enzymes that catalyze creatine synthesis from arginine, glycine and S-adenosyl-methionine [34]. In those rare conditions, usual dietary creatine is not sufficient to meet the need for creatine by the tissues, and severe symptoms occur [35]. Dietary supplementation can then replenish creatine stores, but much higher amounts than usual are needed, up to 800 mg/Kg/day for an infant or 10 g/day for an adult [36], compared to the 1–2 g that are usually obtained through the normal diet [37].

Shewmon and Craig [38] were the first to note that the myopathy induced by statin is characterized by an increased urinary creatine–creatinine ratio. Since in people with normal renal function urinary creatinine is proportional to intramuscular creatine, they postulated that this high urinary creatine—creatinine ratio indicates a deficiency in intramuscular creatine. Although Shewmon and Craig did not actually measure intracellular muscular creatine, later research provided significant support to their assumption.

There is, in fact, evidence that statins administration reduces creatine synthesis. In liver cells, atorvastatin decreases the expression of GAMT (the enzyme that catalyzes the second and final reaction in the synthesis of creatine), leading to reduced intracellular content of creatine [39]. Moreover, a polymorphism of the enzyme glycine amidinotransferase (GATM or AGAT, the enzyme that catalyzes the first step in the synthesis of creatine) is associated with a reduced incidence of statin myopathy [40]. Based on the latter finding, it has been suggested that GATM (also known as AGAT) represents a critical mechanism for the genesis of statin myopathy [41]. Although the association between the GATM polymorphism and statin myopathy was challenged [42,43], Mangravite et al. still maintained that the association they found was significant after adding the new data to their original analysis [44]. It should be noted, however, that these authors did not investigate the functional significance of the polymorphism; specifically, they did not investigate if it was associated with altered levels of intracellular creatine.

## 4. Functions of Creatine in the Muscle

Creatine is essential for normal muscular function. Within the muscle, creatine is phosphorylated to phosphocreatine (PCr). The reaction is reversible, and the two molecules are in constant equilibrium. When phosphocreatine reverts to creatine, its phosphate bond is broken and 45 kJ/mol of free energy become available. By comparison, the phosphate bond that is broken during the conversion from adenosine triphosphate to adenosine diphosphate contains only 31.8 kJ/mol of free energy [34]. Thus, phosphocreatine can transfer its phosphate group to adenosine diphosphate (ADP) in order to resynthesize ATP, a reaction that is catalysed by the creatine-kinase enzyme [34]. In this way, phosphocreatine allows ATP synthesis from ADP along a pathway different from glycolysis. The muscle exploits this unique property of the creatine-phosphocreatine system in two ways (as other cells do too).

**Figure 1:**
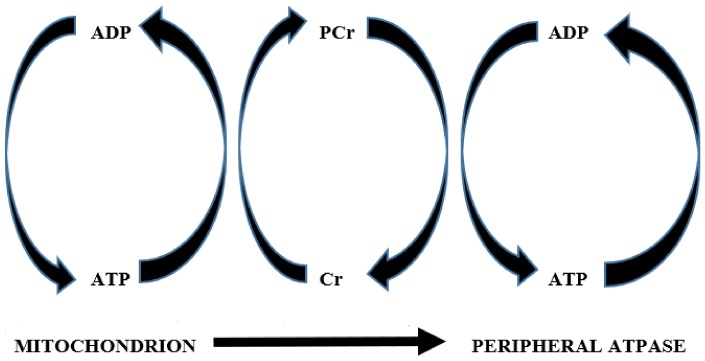
The “ATP shuttle” role of the creatine-phosphocreatine system. In the mitochondrion, oxidative phosphorylation leads to the production of ATP from ADP. The former should travel a considerable length into the cytoplasm to reach the peripheral ATPases enzymes that it must fuel. However, ATP is a rather large and electrically charged molecule; thus, such diffusion would not be easy. Therefore, creatine takes up the phosphate of ATP, transforming itself into phosphocreatine. Since phosphocreatine is a smaller molecule than ATP, it diffuses more easily through the cytoplasm, reaching the peripheral ATPases. There it donates its phosphate group to ADP, providing ATP. By doing so, phosphocreatine reverts to creatine and migrates along its diffusion gradient back to the mitochondrion to start the cycle again [48]. Abbreviations: ATP = adenosine triphosphate; ADP = adenosine diphosphate; Cr = creatine; PCr = phosphocreatine.

First, under normal conditions, phosphocreatine rapidly re-synthesizes ATP near the ATPase enzymes that use it. Besides the plasma Na/K-ATPase that maintains the cell membrane resting potential [45], in the muscle two more ATPase enzymes are at work: myosin and the Sarcoendoplasmic Reticulum Ca^2+^ ATPase (SERCA). Myosin uses ATP to cause muscle contraction [46], and SERCA uses it to cause muscle relaxation (by removing calcium ions from the cytosol, pumping it into the lumen of the sarcoplasmic reticulum) [47]. While these three ATPase enzymes are essential for muscle function, phosphocreatine is essential for their smooth functioning, as long as it provides a ready, nearby source of high-energy phosphate capable of rapidly regenerating ATP upon its use [33]. Furthermore, the creatine-phosphocreatine system takes up the phosphate group of ATP when the latter is synthesized in the mitochondria. It then moves rapidly through the cytoplasm, all the way to the periphery where ATP must be synthesized and used. There, it donates its phosphate group to ADP, synthesizing ATP. This process is known as the “shuttle” function of creatine, because creatine actually takes the high-energy phosphate from the mitochondrion and carries it to the peripheral ATPase [48]. It should be remembered that creatine and phosphocreatine are smaller molecules with a smaller negative charge compared to ATP and ADP, and thus their speed of movement through the cytoplasm is greater. They provide a much more efficient way to carry energy from the mitochondria to the periphery [49]. Figure 1 represents the “shuttle” role of the creatine-phosphocreatine system.

One more role of the creatine-phosphocreatine system in muscle contraction is to provide additional ATP at times of maximal effort such as when blood supply of oxygen and glucose becomes insufficient to synthesize the rapidly depleting ATP. Under these conditions, phosphocreatine provides a ready store of extra phosphate, which allows rapid re-synthesis of ATP independently of oxygen and glucose (“energy buffer” action of phosphocreatine) [34,50].

Last but not least, an important role of creatine in muscular physiology is to favour the differentiation of precursor cells into muscle cells, facilitating the maintenance and recovery of muscle trophism [51,52].

Creatine supplementation has been found capable to improve the symptoms of several pathological conditions of the muscle, including muscular dystrophies, mitochondrial cytopathies, inflammatory myopathies, etc. [53,54].

## 5. Decreasing Creatine Content Harms Muscular Function

The role of creatine in maintaining normal muscle function is further supported by the finding that muscles of mice lacking the enzyme AGAT (also known as GATM, essential step for creatine synthesis) show decreased strength and muscular atrophy [55]. These mice had almost no creatine in their muscles and showed several metabolic abnormalities (for example their inorganic phosphate/β-ATP ratio was increased fourfold, suggesting decreased phosphate utilization in the synthesis of ATP). Morphologically, the muscles of these mice showed alterations consisting of lipid droplets and abnormal crystal structures in the mitochondria and a 70% decrease in muscle volume. On the functional side, mice were hypotonic and showed a more than a 70% decrease in their muscular strength. The described changes normalized almost completely upon dietary supplementation with creatine.

Besides, muscles may be depleted of creatine by feeding mice a creatine analog, guanidino-propionic acid (GPA). Under such experimental conditions, the decrease of creatine content in the muscle causes significant changes in the muscular electrical excitability and contraction, as well as decreased strength and atrophy [31,32,56]. For example, creatine-depleted muscles show mitochondria alterations consisting of the appearance of deposits of abnormal material. Upon further examination, the latter turns out to consist of accumulated creatine-kinase [31]. On the functional side, muscles depleted of creatine and subjected to a burst of intense muscular activity show a decrease in maximum isometric tension, rate of tension development and of relaxation [31]. 

Furthermore, lack of creatine has an important yet usually underestimated role in the normal functioning of the mitochondria. In the above-described “shuttle” function of creatine (Figure 1), creatine works as the acceptor of phosphate at the end of oxidative phosphorylation in the mitochondria. In this role, creatine is the kinetically limiting acceptor that controls respiration [57]. Thus, this might well be the mechanism (or one of the mechanisms) through which diminished intramuscular creatine could impair mitochondrial respiration [38].

In conclusion, the evidence we reviewed so far suggests that statin administration may reduce creatine synthesis and decrease its intracellular content. In turn, muscle lacking creatine shows alterations in muscular strength and volume. The latter effects may be due to several mechanisms (see above):

- Decreased levels of phosphocreatine near cytoplasmic ATPase, which is therefore limiting the substrate (ATP) that is readily available for their function.
- Decreased differentiation of myoblasts into myocytes.
- Lack of sufficient creatine to take up the phosphate from ATP in the mitochondria. This may lead to reduced ATP turnover in the mitochondria, which in turn might be the cause of the mitochondrial dysfunction that was often hypothesized to be the cause of statin myopathy (Table 1).

## 6. Statins Reduce Synthesis of ATP in the Muscle

**Figure 2:**
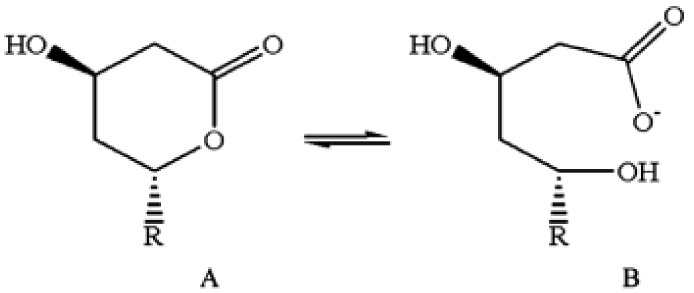
Structure of lovastatin in (**A**) lactone form and (**B**) open hydroxy acid form. After their administration in vivo, all statins exist in both forms, which are at an equilibrium between themselves [63]. Figure reprinted from Patil et al., with permission [64].

From Table 1, it is apparent that mitochondrial damage is often invoked in the pathogenesis of statin myopathy. Mitochondrial dysfunction may harm cells in several ways, including induction of apoptosis through the opening of the mitochondrial permeability transition pore [58,59], and the increased generation by the malfunctioning mitochondria of toxic reactive oxidative species through a “leak” of electrons in the electron transport chain [60,61]. Moreover, reduced production of ATP is certainly a major consequence of mitochondrial dysfunction. Accordingly, when studying the myoblast cell line C2C12 in vitro, Schirris et al. [62] found that almost all the numerous statins they tested decreased maximal ATP production rate, and all their lactone forms did so as well (see Figure 1D of their paper). It should be remembered that some statins are administered as lactone prodrugs, and that all statins interconvert in vivo between lactone and acid form, reaching an equilibrium between these two forms [63] (Figure 2).

Thus, any statin has the potential to decrease ATP production in muscle cells, either by itself or through its lactone form. In the above-quoted experimental investigation [62] all lactone forms proved more effective in reducing maximal ATP production than their acid form. It is interesting to note that the lactone forms of statins in vitro have been found to be more toxic to muscle cells than the corresponding acid forms [65]. Thus, a correlation seems to exist in vitro between statin-induced muscle toxicity and reduction of ATP synthesis.

Still in vitro, levels of ATP were reduced in H9c2 cardiomyocytes after incubation with simvastatin [66].

**Figure 3:**
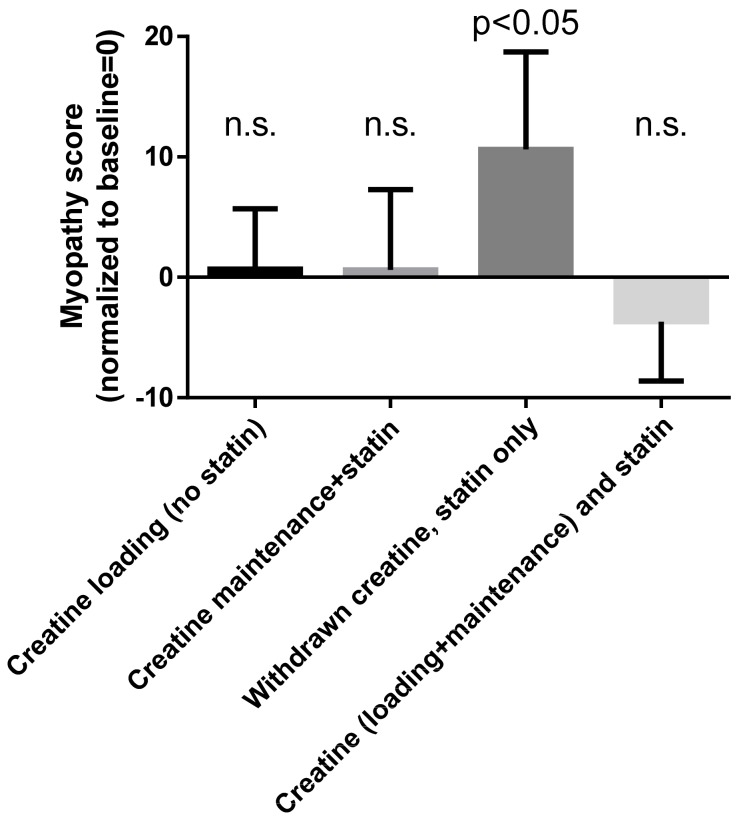
Myopathy score during the various treatments with creatine and/or statin. The graph was designed by us using the data reported by Shewmon and Craig [38]. Statistical findings are for Wilcoxon matched-pairs signed-rank test (2-tailed) comparing each phase with baseline, as reported by Shewmon and Craig; n.s. = not significant. See text for more details.

Furthermore, in the same above-quoted paper [62], Schirris et al. analyzed muscle biopsies from 37 patients with statin-induced myopathies, and found that the mitochondrial ATP production capacity of the muscle was significantly decreased, a finding that remained significant after correction for age and gender (see Figure 3E and Table S2 of their paper).

Thus, one of the major consequences of the mitochondrial impairment that is caused by statins is reduction in cellular ATP.

## 7. Creatine Administration Prevents Statin Myopathy

Some support for the usefulness of creatine supplementation in preventing statin myopathy comes from experimental research, showing that statin treatment facilitates the opening of the mitochondrial transition pore (a signal leading to apoptosis), and that this facilitation is prevented by creatine [67].

At the clinical level, the use of creatine supplementation to prevent statin-associated myopathy has been advocated by Shewmon and Craig [38]. As we reported above, they postulated that a high urinary creatine–creatinine ratio indicates a deficiency in intramuscular creatine, a hypothesis that was later supported by further research [39,40,41,42,43,44].

Starting from this rationale, Shewmon and Craig [38] investigated 12 patients with known intolerance to at least three different statins. For each of them, they calculated a “myopathy score” that took into consideration myalgia, weakness and cramping on visual analog scales. They normalized this score so that at baseline it was zero in each patient. Using a cross-over, open-label study, they withdrew statin treatment, and then they treated each patient with a 5-day loading dose of creatine (5 g twice daily). This loading phase was followed immediately by a 6-week phase during which statin treatment was reintroduced and creatine was administered at a maintenance dose (5 g/day). Then they stopped creatine while continuing the statin until the onset of muscle-toxicity symptoms. Finally, they kept administering statin while reintroducing creatine (loading and maintenance dose, as above). Two patients withdrew from the study for unrelated causes (arthritis and chest pain, respectively). For the remaining patients, the myopathy score was 0.7 ± 5 (mean ± SD) during the initial loading dose of creatine (no statin administration). It remained 0.6 ± 6.7 during the maintenance dose of creatine associated with statin administration. It rose sharply to 10.6 ± 8.1 during the period of statin-only treatment (no creatine) and dropped again to −3.7 ± 4.9 after reintroducing creatine while continuing the statin. Figure 3 summarizes these findings. As we see, at baseline, patients were free from symptoms of myopathy (they had stopped statin administration due to intolerance). They remained symptoms-free during creatine loading (no statin) and creatine maintenance (with added statin). Myopathy relapsed when creatine was stopped (statin only) and again remitted after the reintroduction of creatine, despite continuing statin (creatine and statin). Wilcoxon’s test showed no significant differences between all these values and the baseline except for the statin-only (no creatine) phase (*p* < 0.05).

**Figure 4:**
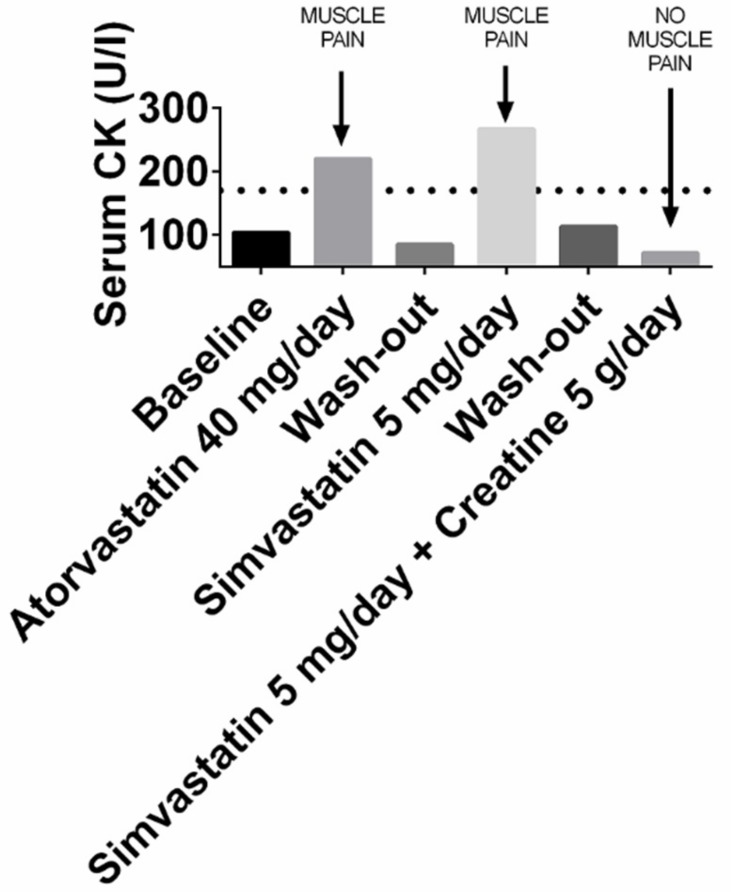
Serum levels of creatine kinase (CK) and muscle pain in the patient we treated with creatine supplementation. Muscle pain occurred and CK levels rose to abnormal levels when statins were prescribed, but not when the statin was prescribed together with creatine. Reprinted from reference [68], with permission of the publisher.

Quite surprisingly, the paper by Shewmon and Craig had no follow up, and creatine treatment of statin myopathy was, to the best of our knowledge, no longer investigated until we recently decided to treat one such case with creatine supplementation [68]. We cured a 66 year old lady who had shown muscle pain and serum creatine kinase elevation twice, after treatment with either atorvastatin 40 mg/day or simvastatin 5 mg/day. Since her LDL-cholesterol was off-target and she had a significant cardiovascular risk (carotid stenosis and an episode of amaurosis fugax), statin treatment was mandatory. Thus, we treated her with creatine supplementation and found, in agreement with the data by Shewmon and Craig, that the same simvastatin dose that had earlier caused intolerance was now well tolerated. Figure 4 (reprinted from our original paper, with permission of the publisher) summarizes this patient’s findings. Although we could not derive any statistics from this single patient, the results of our crossover treatment is suggestive of efficacy, and it is consistent with Shewmon and Craig’s findings.

## 8. Discussion and Conclusions

Preclinical evidence shows that creatine treatment prevents the harmful effects of statins on the mitochondria [67].

Although the number of treated patients is limited, two clinical papers [38,68] show promising results in creatine treatment of statin myopathy. Both had a cross-over design, meaning that the same patients were studied both with and without creatine supplementation, and both showed that the same patients were intolerant to statins at baseline, but were no longer intolerant after supplementation with creatine.

The rationale for this effect of creatine may be that it may correct a decrease in the creatine content of statin-treated cells [39]. Such decrease might indeed be the cause of the mitochondrial malfunction that many authors hypothesized as a cause of statin myopathy. In fact, as Shewmon and Craig originally noted [38], creatine is the kinetically limiting acceptor that controls respiration, and thus diminished intramuscular creatine could impair mitochondrial respiration [57].

We acknowledge that further research should be done on these subjects. For example, muscle creatine in statin-induced myopathy should be measured to possibly confirm its decrease. In fact, so far the only investigation that was carried out on this topic was done in liver cells [39]. Albeit it was positive (it found that atorvastatin did indeed decrease creatine content), it certainly needs confirmation in muscle cells or tissue. Furthermore, it should be noted that the fact that in statin myopathy there is a high urinary creatine–creatinine ratio [38] does not per se indicate a decrease of creatine in the muscle. In theory, it might indicate either increased blood plasma creatine linked to higher excretion rate and/or lower formation and excretion of creatinine. Nevertheless, and pending future studies, the above findings suggest that creatine supplementation may be a simple way to prevent statin-induced myopathy.

Additional clinical trials should be carried out to hopefully provide further and more conclusive evidence on the usefulness of creatine in statin myopathy. However in the meantime, we emphasize that creatine is a legally available, widely used dietary supplement, and that double-blind, placebo-controlled trials have demonstrated its safety even in people of a more advanced age [36,69,70,71]. Thus, we believe that, in view of its safety and easy availability, creatine supplementation should be trialed, on a case-by-case basis and under medical supervision, in those patients at risk for cardiovascular diseases whom statin myopathy prevents from reaching their cholesterol goals.

## Figures and Tables

**Table 1:** Recent hypothesis on the pathogenesis of statin-induced (not autoimmune) myopathy.

Paper	Mechanisms Proposed
Tomaszewski et al., 2011 [24]	Altered membrane function due to lower cholesterol content. Altered mitochondrial function due to decreased muscle coenzyme Q10 (CoQ10). Impairment of calcium homeostasis. Induction of apoptosis. Genetic determinants.
Vrablik et al., 2014 [25]	Decreased intracellular concentrations of cholesterol. Reduced production of coenzyme Q10 and related ubiquinones. Decreased production of prenylated proteins. Increased uptake of cholesterol from the extracellular space. Increased uptake of phytosterols. Disruption of calcium metabolism in myocytes. Decreased renewal of damaged muscle cells via the ubiquitin pathway. Inhibition of selenoprotein synthesis. Genetic factors ^1^. Unmasking of pre-existing muscular disorders
Apostolopoulou et al., 2015 [26]	Impairment of mitochondrial function. Decreased muscle coenzyme Q10 (CoQ10). Genetic susceptibility.
Laufs et al., 2015 [27]	Reduction of cholesterol/isoprenoid concentrations in specific cellular and subcellular compartments. Reduced sarcolemmal and/or sarcoplasmic reticular cholesterol. Alterations of myocellular fat and/or sterol concentration. Increased catabolism of muscular proteins or decreased catabolism of damaged proteins. Failure to repair damaged muscle. Leakage of sarcolemmal calcium into the cytoplasm. Impairment of mitochondrial function ^2^.
Muntean et al., 2017 [28]	Increased fatty acid synthesis and induced triacylglycerol and phospholipid accumulation in lipid droplets ^3^. Inhibition of the mevalonate pathway and subsequent decrease in availability of isoprenoid intermediates, leading to decreased synthesis of cholesterol, ubiquinone and dolichols, and to impaired prenylation of structural proteins. Calcium release from sarcoplasmic reticulum and mitochondria. Impairment of oxidative phosphorylation. Decrease in mitochondria density and biogenesis. Apoptosis and calpain-mediated cell death. Impairment of muscle regeneration and the remodeling of cytoskeletal architecture.
du Souich et al., 2017 [29]	Increased statin accumulation in the myocyte, resulting from the reduced function of the transporters carrying statins into cells or their metabolites out of them. Altered mitochondrial function causing reduced production of ATP, excess production of reactive oxygen species (ROS) and apoptosis. Reduced ubiquinone levels. Toxic effect of statins on mitochondrial function. Direct effect of statins on sarcoplasma chloride and lactate.
Selva-O’Callaghan et al., 2018 [30]	Mitochondrial dysfunction. Oxidative stress. Impaired mevalonate metabolism. Isoprenylation of small G-proteins. Genetic susceptibility (polymorphisms of the SLCO1B1 gene ^4^, alterations in genes coding for plasma membrane calcium transporting ATPase and alterations of the CoQ2 gene ^5^) ^6^.

^1^ Twenty-seven suspected genes are listed, including the gene encoding for the precursor of creatine guanidine acetic acid (GAA) and the genes ATP1A1, ATP1A2 and ATP1B1 encoding for the α_1,_ α_2_ and β_1_ subunits, respectively, of Na/K-ATPase. ^2^ These authors list the autoimmune mechanism too, apparently not making a clear distinction between autoimmune-mediated effects of statins and their direct toxic or metabolic effects. ^3^ This effect of losuvastatin was found in cultured cells in vitro, and the authors remain unsure whether or not it affects clinical toxicity. ^4^ The solute carrier organic anion transporter family member 1B1, which is responsible for the entry of statins into cells. ^5^ Coding for coenzyme Q10. ^6^ These authors list the anti-HMCGR autoimmune mechanism too, apparently not making a clear distinction between autoimmune-mediated effects of statins and their direct toxic or metabolic effects.
